# Using a Smartphone-Based Colorimetric Device with Molecularly Imprinted Polymer for the Quantification of Tartrazine in Soda Drinks

**DOI:** 10.3390/bios13060639

**Published:** 2023-06-09

**Authors:** Christian Jacinto, Ily Maza Mejía, Sabir Khan, Rosario López, Maria D. P. T. Sotomayor, Gino Picasso

**Affiliations:** 1 Laboratory of Instrumental Analysis Environment, Faculty of Sciences, National University of Engineering, Av. Tupac Amaru 210, Rimac 15333, Lima, Peru; christian@uni.edu.pe (C.J.); imaza@uni.edu.pe (I.M.M.); sabir@ufersa.edu.br (S.K.); rclopez@uni.edu.pe (R.L.); 2 Technology of Materials for Environmental Remediation Group (TecMARA), Faculty of Sciences, National University of Engineering, Av. Tupac Amaru 210, Rimac 15333, Lima, Peru; gpicasso@uni.edu.pe; 3 Chemistry Institute-Araraquara-SP, São Paulo State University (UNESP), Araraquara 14801-900, Brazil; 4 Department of Natural Sciences, Mathematics, and Statistics, Federal Rural University of the Semi-Arid, Mossoro 59625-900, Brazil

**Keywords:** smartphone, molecularly imprinted polymers (MIP), tartrazine, colorimetric detection, UHPLC

## Abstract

The present study reports the development and application of a rapid, low-cost in-situ method for the quantification of tartrazine in carbonated beverages using a smartphone-based colorimetric device with molecularly imprinted polymer (MIP). The MIP was synthesized using the free radical precipitation method with acrylamide (AC) as the functional monomer, N,N′-methylenebisacrylamide (NMBA) as the cross linker, and potassium persulfate (KPS) as radical initiator. The smartphone (RadesPhone)-operated rapid analysis device proposed in this study has dimensions of 10 × 10 × 15 cm and is illuminated internally by light emitting diode (LED) lights with intensity of 170 lux. The analytical methodology involved the use of a smartphone camera to capture images of MIP at various tartrazine concentrations, and the subsequent application of the Image-J software to calculate the red, green, blue (RGB) color values and hue, saturation, value (HSV) values from these images. A multivariate calibration analysis of tartrazine in the range of 0 to 30 mg/L was performed, and the optimum working range was determined to be 0 to 20 mg/L using five principal components and a limit of detection (LOD) of 1.2 mg/L was obtained. Repeatability analysis of tartrazine solutions with concentrations of 4, 8, and 15 mg/L (*n* = 10) showed a coefficient of variation (% RSD) of less than 6%. The proposed technique was applied to the analysis of five Peruvian soda drinks and the results were compared with the UHPLC reference method. The proposed technique showed a relative error between 6% and 16% and % RSD lower than 6.3%. The results of this study demonstrate that the smartphone-based device is a suitable analytical tool that offers an on-site, cost-effective, and rapid alternative for the quantification of tartrazine in soda drinks. This color analysis device can be used in other molecularly imprinted polymer systems and offers a wide range of possibilities for the detection and quantification of compounds in various industrial and environmental matrices that generate a color change in the MIP matrix.

## 1. Introduction

Tartrazine (TAR) is a highly popular yellow dye that is widely applied in a variety of food and beverage products, including sweets, juices, and general food products. Several studies reported in the literature have raised serious concerns regarding the potential health risks associated with the use of TAR in food products; these studies have linked Tartrazine to a wide range of negative health problems, including headaches, neurotoxicity, genotoxicity, and even cancer. Bearing that in mind, consumers are advised to take note of the potential risks associated with tartrazine and consider limiting their intake of products that contain this dye [1]. The recommended daily intake of tartrazine in humans ranges between 0 and 7 mg/L5 mg/kg of body weight [2]. Azo dyes, such as tartrazine, are known to undergo reductive biotransformation during metabolism, and this can lead to the release of toxic and potentially carcinogenic compounds in the body. The presence of these compounds in the human body affects the DNA, as it causes mutagenic effects, and increases the risk of cancer [3]. Therefore, regulating the levels of tartrazine in food products is essentially crucial as it helps to ensure food safety and protect public health [4].

Several studies have proposed a plethora of techniques for the determination of tartrazine. High-performance liquid chromatography (HPLC) is the technique most widely applied for TAR determination due to its high sensitivity and selectivity [5]; however, this technique is found to be costly and its application often requires the use of significant amounts of toxic solvents [6,7]. Spectrophotometric methods are also widely used for TAR determination, though their specificity may be limited due to spectral overlap with other species in the matrix [8,9]. Among the more recent analytical techniques that have been applied for TAR determination are paper chromatography [10] and voltammetry [11,12]. A comprehensive outline of other methods used for TAR determination in food products can be found in Rovina’s work [13].

Molecularly imprinted polymers (MIPs) are synthetic analogs of biological antibody-antigen systems [14]; these materials function via a “lock and key” mechanism with the selective binding of the molecule to which they are designed during synthesis. MIPs offer the potential specificity and selectivity of biological receptors with the explicit advantages of durability under environmental conditions and at low costs [15]. MIPs can be employed for a wide range of purposes, including the separation and purification of chemical compounds [16,17,18], catalysis [19], detection and quantification of substances in complex samples [20,21,22], and in the fabrication of sensory and biosensor devices [23,24,25]. MIPs can also be used as optical sensors for dyes, where they modulate the changes in the optical properties of the polymer upon binding to the dye. For this reason, they have been used for the determination of textile [26,27] and food colorants [28,29,30].

The use of smartphones in digital color analysis is an innovative technique that allows for accurate and easy measurement and effective comparison of the color of different objects or surfaces [31,32]. Under this technique, the smartphone camera is used to capture the images of colored objects which are intended to be analyzed. In addition, image processing algorithms are applied aiming at extracting the numerical color values in different color spaces, including the following: Red, green, blue (RGB); cyan, magenta, yellow, and key (black) (CMYK); hue, saturation, value (HSV); and CIELAB [33]. Specific image processing software, such as Adobe Photoshop, Image-J, MATLAB, etc. are used for color quantification under the appropriate color space. A relationship between the quantified image data and the analyte concentration can then be established using multivariate calibration models, such as partial least-squares (PLS) [34] and machine learning [35]. Currently, the widespread use of mobile applications (apps) and the high computing power of smartphones have led to an increasingly growing application of these devices in digital color analyses for point-of-care testing (POCT) [32]. POCT involves the conduct of rapid and simple sample detection tests using a portable instrument at the sampling site which helps in significantly reducing the time and cost of the test [36]. This testing technique has been used in various areas, including clinical diagnosis [37,38], food safety [39,40], environmental testing [41,42], among others [43,44].

Previous works reported in the literature have employed MIP for tartrazine determination analyses. Arabzadeh [45] synthesized and used MIP for tartrazine adsorption. Córdova [29] reported the use of modified MIP in core-shell type nanoparticles for tartrazine determination in carbonated beverages through reflectance. This work is the first to use MIP for colorimetric quantification of tartrazine using a smartphone. Other similar works have been reported to determine antibiotics, such as sulfamethoxazole [46], fungal toxins, such as aflatoxin B1 [47], and dyes, such as malachite green [48], basic red 9 [49], and Erythrosine B [50]. In our present work, MIP will be used for tartrazine determination in soda drinks through the application of color analysis. To conduct this analysis, an image capture device (RadesPhone) which uses a smartphone camera will be constructed, and the image of the MIP exposed to aqueous samples of tartrazine will be obtained. After obtaining the digital image of the MIP, the appropriate software will be used to extract the values of RGB, HSV, L, and I color channels, and PLS multivariate regression analysis will be conducted to determine tartrazine in the samples. The application of color analysis with the constructed device opens up a wide range of possibilities for the use of different types and forms of MIP, as well as for the rapid detection and quantification of different compounds in industrial and environmental matrices at low-cost and without the need of specialized personnel.

## 2. Materials and Methods

### 2.1. Chemic als and Reagents

All reagents used in this work were of analytical grade. Solutions were prepared in ultrapure water (18 MΩ at 25 °C). Tartrazine (TAR), N,N′-methylenebisacrylamide (NMBA), acrylamide (AA), and potassium persulfate (KPS) were purchased from Sigma-Aldrich. Sodium hydroxide, methanol (HPLC grade), concentrated ammonia solution (23–28%), and hydrochloric acid (10%) were purchased from Merck.

The infrared spectrum was obtained using a Shimadzu IRPrestige 21 in ATR mode to show the functionalization and polymerization of the MIP through the presence of different functional groups. The light intensity measurement was performed with luxmeter UT383 Mini Light Meters. UHPLC was performed using a Thermo Scientific™ Vanquish™ Duo UHPLC system with a C18 column of 150 × 4.6 mm, UV detector, and particle size of 5 μm.

### 2.2. Synthesis of Molecularly Imprinted Polymer (MIP)

The MIP was prepared based on the precipitation method using the general synthesis procedure proposed by Arabzadeh [45], but it was modified to a molar ratio of template, functional monomer, and cross-linking agent of 1:2:100, respectively. For this, 0.1 mmol of tartrazine (template) and 0.2 mmol of acrylamide (functional monomer) were mixed in 40 mL of ultrapure water (porogenic solvent) and remained under agitation for 2 h. The solution was bubbled with N_2_ for 10 min to remove oxygen, and then 10 mmol of N,N′-methylenebis (acrylamide) (cross linker) was added. Next, 0.185 mmol of potassium persulfate (initiator) was added, and the solution was continuously treated with nitrogen for another 10 min. Finally, the reaction flask was placed in a water bath at 60 °C for 22 h under constant agitation. After obtaining the MIP, tartrazine was extracted using a Soxhlet extraction system with an 8:2 (*v*/*v*) water/ammonia solution until the washing solutions showed no UV signal of tartrazine. The obtained material was washed with a mixture of ethanol and water, dried at room temperature in a vacuum desiccator for 1 day, ground in an agate mortar, and passed through a 250 μm sieve (No. 60).

### 2.3. Adsorption Process

To evaluate the effect of pH and adsorption time, the MIP prepared under the new conditions was used. To adjust the procedure and obtain an amount of MIP that could be used in image analysis, 15 mg of MIP and 15 mL of tartrazine sample at pH 3.0 were placed in a 20 mL flask, agitated in a homogenizer for 10 min and filtered through Whatman No. 40 filter paper. The solid was removed from the paper and dried in an oven at 60 °C to make it available for image analysis.

### 2.4. Construction of the RadesPhone Device

**Figure 1:**
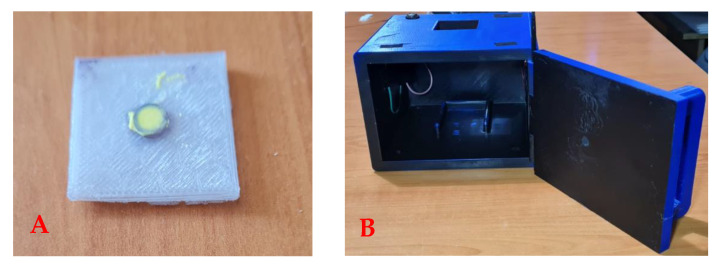
RadesPhone device for image acquisition. (**A**) Sample holder with dimensions of 3 × 3 cm and height of 3 mm; (**B**) camera for image acquisition.

Both the sample holder and the imaging device were constructed using a 3D printer. The sample holder was a 3 × 3 cm platform with a thickness of 3 mm, and it had a space for the solid sample in the center (Figure 1A). The RadesPhone imaging device was constructed with dimensions of 15 × 15 × 10 cm and was completely painted black on the inside (Figure 1B). A previous study determined that the dimensions of the imaging device and the height of the smartphone did not influence image capture [49]. Two arrays of LED lamps were placed at the top corners of the device providing a light intensity of 170 lux, which operated by connecting them to the power outlet.

### 2.5. Image Acquisition with RadesPhone

**Figure 2:**
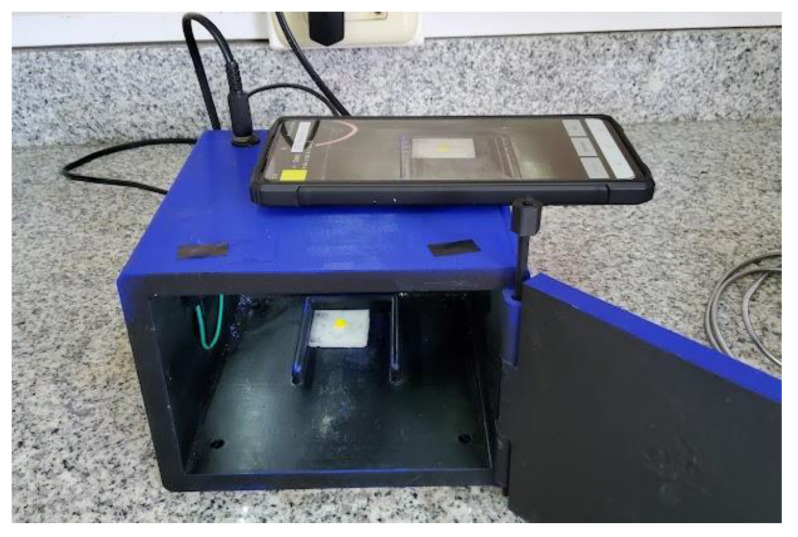
RadesPhone device with the smartphone for imaging the MIP on the sample holder.

A Samsung Galaxy S20 FE smartphone, model SM-G780F, with a 12 MP rear camera was used for image capture. The image capture settings were adjusted with the camera sensor sensitivity set to automatic ISO, a camera shutter speed of 1/60 s, an exposure EV of 0, and an automatic white balance. The sample holder containing the MIP exposed to tartrazine solutions was placed in the corresponding RadesPhone imaging device for image capture (Figure 2).

### 2.6. Analysis of Soda Drinks by UHPLC

The reference quantities of tartrazine in the carbonated beverages were quantified using UHPLC measurements as described above. The mobile phase consisted of a mixture of methanol and water (20:80 *v*/*v*), with a flow rate of 1 mL/min, a wavelength of 430 nm, and an injected sample volume of 20 μL.

## 3. Results

### 3.1. Synthesis of MIP Obtained by Precipitation

The MIP prepared by Arabzadeh [45] presented the characteristic yellow color of tartrazine, even after being washed for a week. This background color reduces the sensitivity of the MIP for its use in subsequent color analysis when exposed to tartrazine solutions. To improve the sensitivity of the MIP, it was proposed to modify the template:MF:ME molar ratio to 1:2:100. This proportion increases the amount of ME in the MIP synthesis, which produces a significant decrease in the intensity of the yellow color of tartrazine in the resulting MIP. This new MIP was employed in tartrazine adsorption studies for subsequent color analysis using a smartphone.

**Figure 3:**
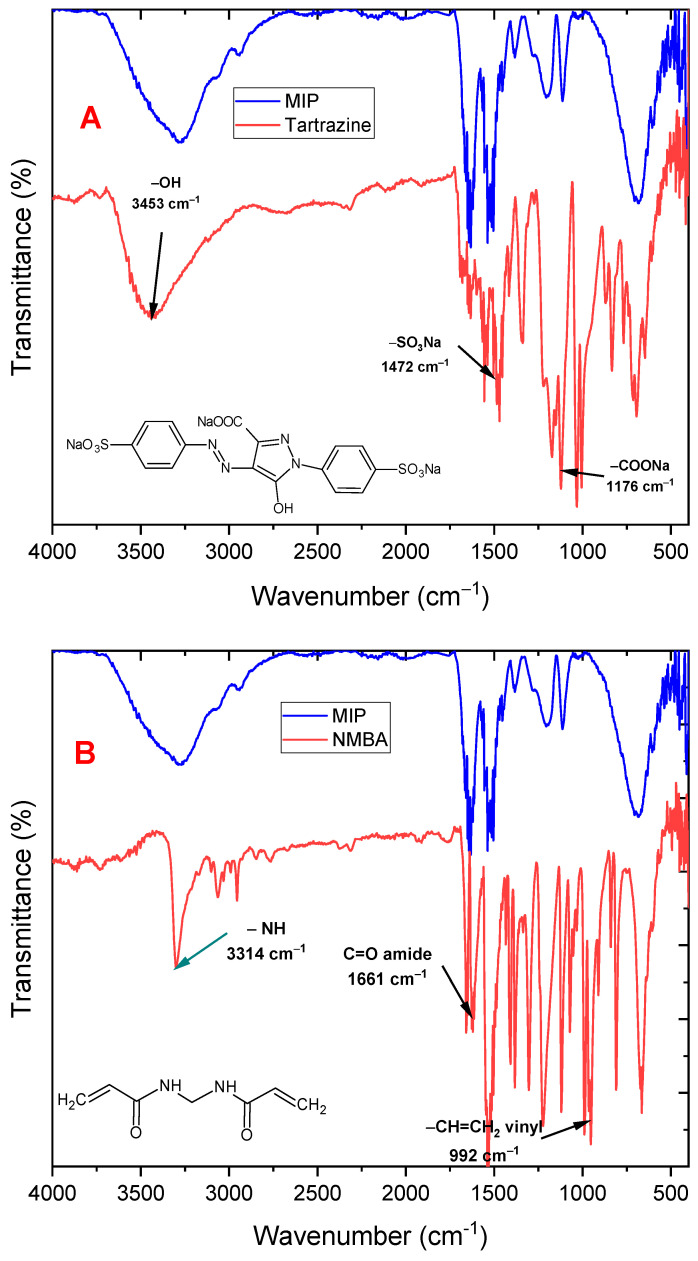
ATR-mode FTIR spectra of (**A**) MIP and tartrazine, (**B**) MIP and N,N′-methylbisacrylamide cross-linking monomer.

Some characterization tests were performed on the newly synthesized MIP to verify that the MIP was obtained, as well as some tartrazine adsorption tests in aqueous solutions. Figure 3A shows the infrared spectra of the MIP and tartrazine, displaying the characteristic peaks of the polymer at 1675 cm^−1^ and 3342 cm^−1^, corresponding to the C=O and -NH- vibration peaks, respectively. Additionally, tartrazine exhibits peaks at 3453, 1472, and 1176 cm^−1^ corresponding to the hydroxyl (-OH), sulfonate (-SO_3_Na), and carboxylate (-COONa) linkages, respectively, and shows that the MIP does not exhibit appreciable signals of tartrazine. Figure 3B compares the spectra of the MIP with N-N′-methylene-bis-acrylamide (cross linker). The C-H doublet peak of the out-of-plane deformation of the vinyl group (992 and 910 cm^−1^) disappears in the MIP, indicating that polymerization was carried out through the C=C double bond of this monomer. The C=O and -NH peaks of the monomer (at 1661 cm^−1^ and 3314 cm^−1^) remain in the MIP since the cross linker is present in a higher proportion in the MIP.

**Figure 4:**
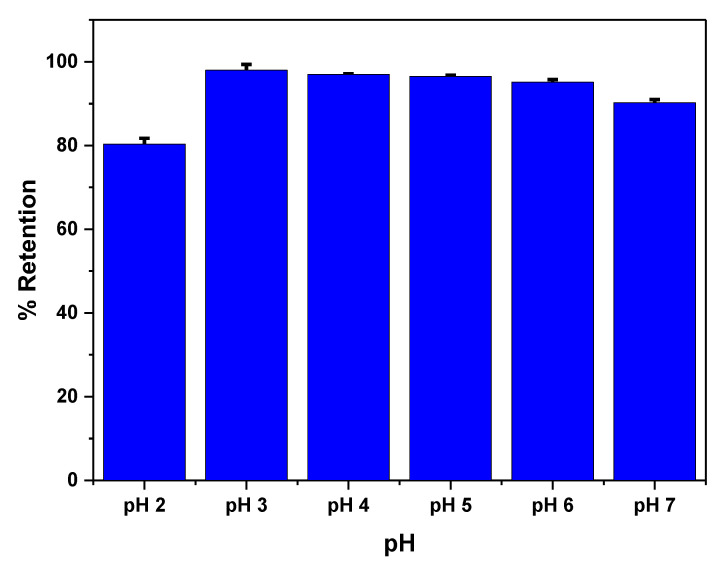
Effect of pH on tartrazine removal. Measurements were made using 6 mg of MIP with 2 mL of tartrazine solution and stirred for 1 h.

The effect of pH is studied to achieve the maximum adsorption capacity of the polymer (Figure 4). It has been observed that the maximum adsorption occurs at a pH of 3.0. This preference for adsorption at pH 3.0 is due to the fact that the sulfonate group of the polymer has a pKa of 3.2, indicating that it is in its ionic form at this pH. Under these conditions, the interaction between tartrazine and the MIP primarily occurs through hydrogen bonding and electrostatic interactions. The electrostatic interactions are established between the non-dissociated amide group present on the polymer surface and the sulfonate group of tartrazine [51].

P − NH_2_ + H^+^ ↔ P − NH_3_^+^

T − SO_3_Na → T − SO_3_^−^ + Na^+^

T − SO_3_^−^ + P − NH_3_^+^ → T − SO_3_^−^ + NH_3_ − P

Molecular imprinting also has an effect on the adsorption capacity of the MIP for tartrazine. This effect has been evaluated by studying the selectivity of the MIP compared to another similar dye, such as Sunset Yellow CFC. In a study conducted by Arabzadeh [45], the selectivity factor of the MIP for tartrazine relative to Sunset Yellow CFC was determined to be 22.5, while the NIP showed a value of 0.73. These results highlight the impact of molecular imprinting on the selective adsorption of tartrazine.

**Figure 5:**
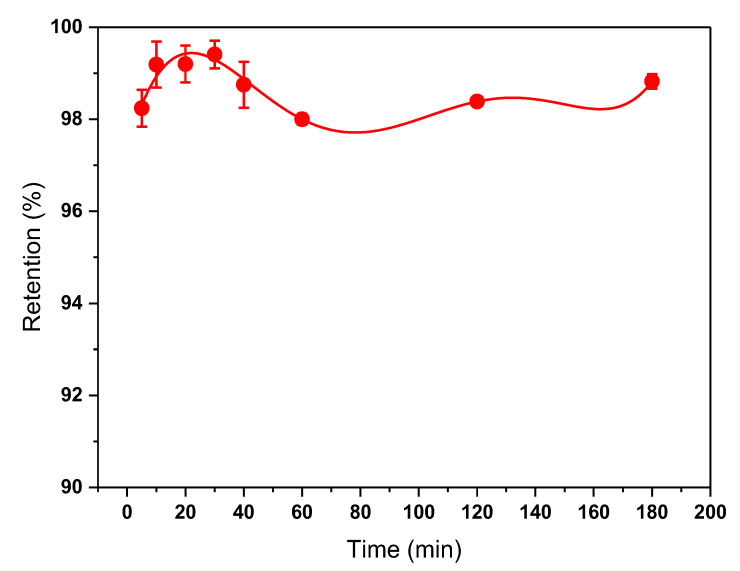
Effect of MIP contact time on tartrazine adsorption. Measurements were made using 6 mg of MIP with 2 mL of tartrazine solution at pH 3.0.

The effect of contact time of MIP on tartrazine adsorption was evaluated at different times up to 3 h and is shown in Figure 5. The adsorption rate of tartrazine was rapid initially, with maximum adsorption achieved at 10 min. It was also observed that at contact times greater than 30 min, desorption of tartrazine occurred, resulting in a decrease in % removal, although after 60 min it increased again.

**Figure 6:**
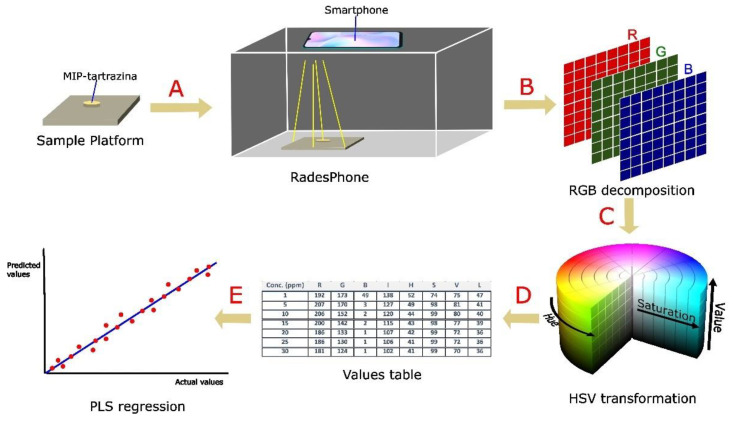
General scheme of color analysis for the determination of tartrazine using MIP. (**A**) Capturing 16 × 16 pixel image of MIP with tartrazine; (**B**) decomposition of the image into RGB; (**C**) transformation to HSV, L, and I color channels; (**D**) obtaining the table of values of the color channels; (**E**) multivariate regression with the values of the channels obtained from each sample.

The general procedure for color analysis using the RadesPhone device is as follows. The MIP exposed to the tartrazine samples is filtered and dried in an oven at 60 °C. Subsequently, it is placed in the sample holder along with the RadesPhone device (Figure 6). The digital image of the sample was obtained using the smartphone camera. With the assistance of the free software Image-J, we obtained the RGB values from a 16 × 16 pixel region in the image. By converting these values to the other channels (using Equations (1)–(5)), we obtained the corresponding HSV, L, and I values. With the values of these channels, we perform a multivariate regression PLS and select the number of components that provide the lowest prediction error. Moreover, we select the working range and evaluate the repeatability and detection limit, as well as the application to soft drink samples.

Since RGB values are not the only ways to represent color, conversion to other channels is performed. The H, S, and V channels represent the hue, saturation, and value components of an image. The hue channel describes the color tone, while saturation refers to the amount of color present in the image, and the value describes the intensity or brightness of the color. The L channel is a component of the CIELAB color space used to measure color differences perceived by the human eye and describes the lightness of a color. The I channel refers to the intensity component in the HSI (hue, saturation, intensity) color space, which is often used to describe the image in terms of color and brightness. RGB values are obtained from the image using Image-J. The other HSV, L, and I values are calculated from Equations (1)–(5):

(1) $$H=\left\{\begin{array}{c} undefined,\;\;\;\;if\;\max=min\\ 60{}^{\circ}\times \frac{G-B}{\max-min}+0{}^{\circ},\begin{array}{c} if\;max=R\\ and\;G\geq B \end{array}\\ 60{}^{\circ}\times \frac{G-B}{\max-min}+360{}^{\circ},\begin{array}{c} if\;\max=R\\ and\;G<B \end{array}\\ 60{}^{\circ}\times \frac{B-R}{\max-min}+120{}^{\circ},if\;\max=G\\ 60{}^{\circ}\times \frac{R-G}{\max-min}+240{}^{\circ},if\;\max=B \end{array}\right.$$

(2) $$S=\left\{\begin{array}{c} 0,\;\;\;\;if\;\max=0\\ 1-\frac{min}{max},\;\;\;in\;another\;case \end{array}\right.$$

(3) V=max

(4) $$L=\frac{1}{2}\left(\max+min\right)$$

(5) $$I=\frac{R+G+B}{3}$$

These values can be obtained from websites, such as https://www.peko-step.com/es/tool/hslrgb.html. A detailed explanation of what each color channel represents in its color space is described in the literature [34,52,53].

The RGB, HSV, L, and I values obtained for the standards and samples are presented in Table 1:

### 3.2. Multivariate PLS Regression

**Figure 7:**
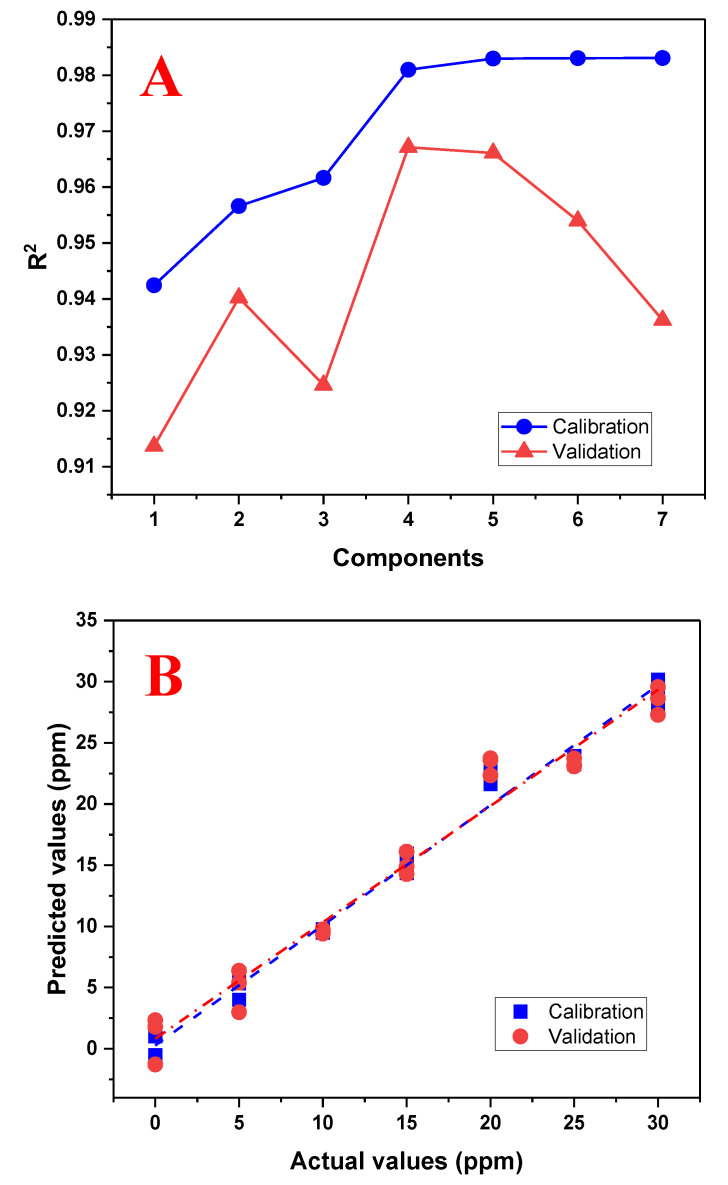
Performance graphs of the PLS regression model using the range of 0 to 30 mg/L of tartrazine. (**A**) Graph of the number of principal components used with the R^2^ calibration and validation; (**B**) graph between the actual (real) and predicted (calculated) values by the PLS model using four principal components.

PLS is a multivariate calibration method that seeks factors that explain the highest possible covariance between a set of predictor variables, X, and response variables, Y. A training matrix was developed consisting of 21 samples with concentrations of 0, 5, 10, 15, 20, 25, and 30 mg/L of tartrazine at pH 3.0, prepared in triplicate. PLS first performs a principal component analysis (PCA) to reduce the dimensionality of the original variables. A regression model is then developed using these principal components. Cross-validation is used to evaluate the performance of the model, which assesses the ability of the PLS model to predict the responses of a data sample that was not used in calibration. To evaluate the number of principal components that provide the best prediction, a graph was plotted showing the use of these components with the coefficient of determination R^2^ between the measured value and the predicted value. The number of components that provide the highest R^2^ value will define the number of components to use. Figure 7A shows the graph for selecting the number of principal components, which indicates that four components provide the best regression model. After selecting the four components to use, the PLS model is evaluated by plotting the measured values against the predicted values (Figure 7B). This graph shows that there is no good linear fit beyond 20 mg/L, and thus the working range will be reduced to 20 mg/L.

**Figure 8:**
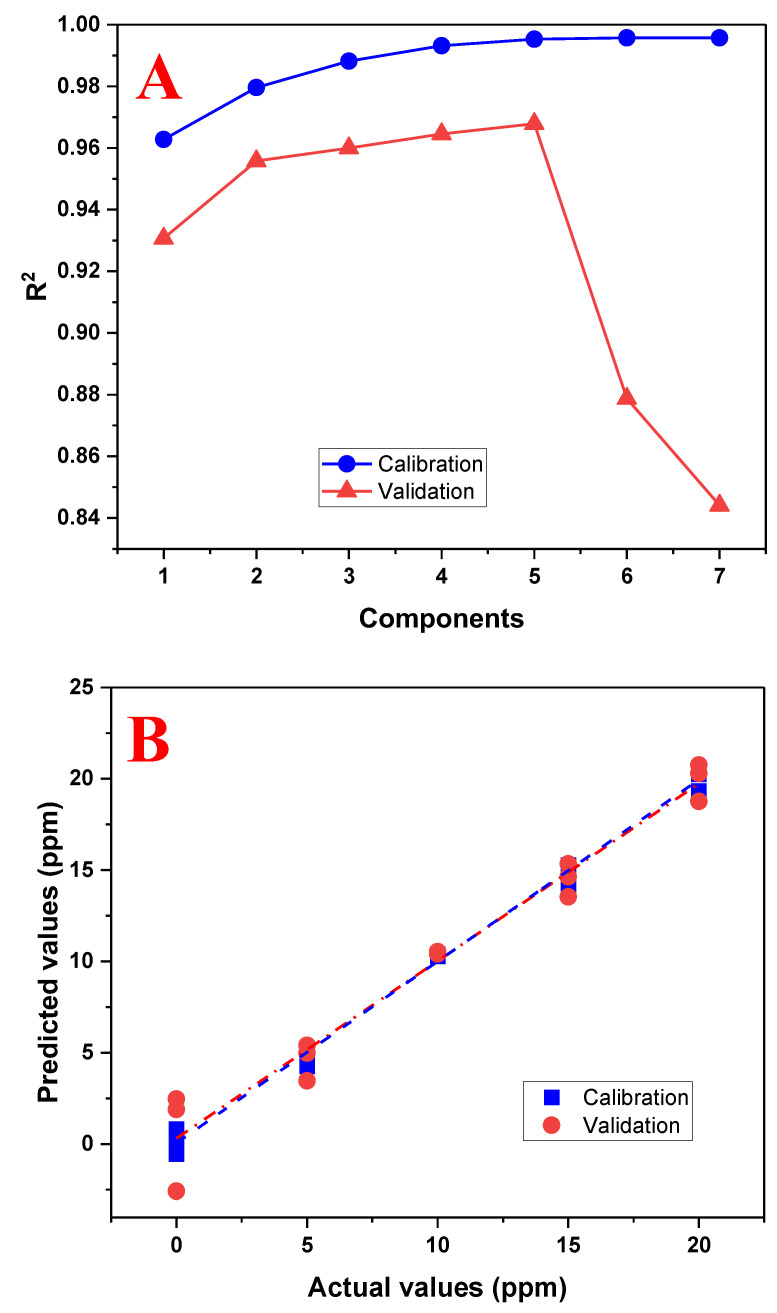
Performance graphs of the PLS regression model using the range of 0 to 20 mg/L of tartrazine. (**A**) Graph of the number of principal components used with the R^2^ calibration and validation; (**B**) graph between the actual (real) and predicted (calculated) values by the PLS model using five principal components.

The PLS model was re-evaluated using the range of 0 to 20 mg/L and the results are shown in Figure 8. It can be seen that the model improved and it will be used for future sample predictions. A summary of the performance results of both evaluated models is shown in Table 2.

Table 3 shows the performance metrics of the obtained PLS model. Repeatability was tested at different concentration levels, showing that variability increases as concentration decreases. The detection limit was calculated based on the standard deviation of the regression of the PLS model between measured and predicted values [54]. This value is related to the initial yellow color of the MIP and, as previously mentioned, the cross linker ratio was modified during synthesis to obtain an MIP suitable for color analysis. The results obtained are satisfactory, demonstrating that color analysis using the RadesPhone device and the MIP for tartrazine determination is appropriate.

**Figure 9:**
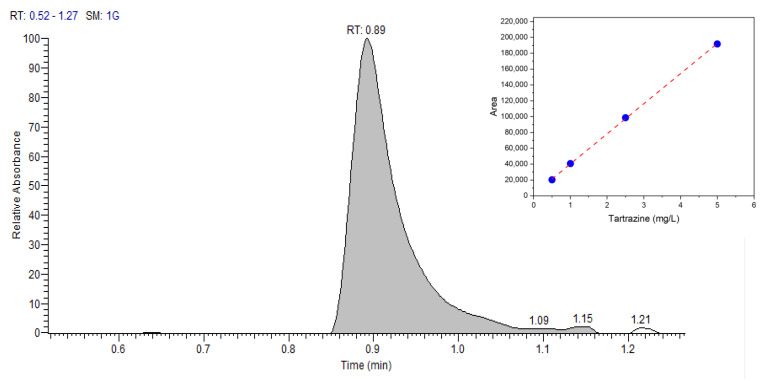
UHPLC chromatogram of 1 mg/L tartrazine, mobile phase methanol and water (20:80 *v*/*v*), flow rate of 1 mL/min, wavelength of 430 nm, and injected sample volume of 20 μL. Thermo Scientific™ Vanquish™ Duo UHPLC system liquid chromatograph with 150 × 4.6 mm C18 column, UV detector, and particle size of 5 μm.

The results of the proposed RadesPhone method were compared to those obtained by UHPLC for real samples of soda drinks. A typical chromatogram and the analytical curve is shown in Figure 9.

The comparison results are shown in Table 4. Despite the low concentrations being worked with, the results are satisfactory. In general, the proposed method’s results are slightly higher (except for M2) than those of the UHPLC reference method, possibly due to the adsorption of other yellow dyes that the samples may contain, such as sunset yellow, which is common in these types of foods.

This method is comparable to the results obtained by Córdova [29] in his study on soda drinks samples. In that study, results were reported with a relative error between 14% and 23% (alternative method between 7.7% and 16%), and a relative standard deviation between 12% and 20% (alternative method between 3.5% and 6.3%).

## 4. Conclusions

In the present work, a color analysis device (RadesPhone) using MIP has been developed for the quantitative analysis of tartrazine in soda drinks. The MIP used in this study was synthesized using the precipitation method and had previously been employed in studies involving the adsorption of tartrazine in food samples. In this work, the same MIP was utilized for both the adsorption of tartrazine and the subsequent color analysis using a smartphone. Color analysis has been demonstrated to be a powerful tool for quantifying tartrazine in carbonated beverages using the RadesPhone device and MIP. The method, which involves transforming the digital image into RGB color values and employing multivariate regression PLS, has demonstrated excellent repeatability and low prediction errors. Comparing the results obtained with a similar study that utilized a modified MIP for tartrazine determination in soda drinks, the proposed method with the smartphone exhibited lower prediction errors (23% relative error in the modified MIP method compared to 16% in the proposed smartphone method) and reduced variability (20% RSD in the similar method compared to 6.3% in the proposed method). The utilization of smartphones in color analysis with the RadesPhone device enables the development of a simple and cost-effective method for determining soda drinks. This approach eliminates the need for solvents or specialized equipment and allows for untrained personnel to perform the analysis, following the principles of point-of-care testing (POCT). The results obtained by color analysis were slightly higher than those obtained by UHPLC, possibly due to interferences present in the color analysis and the higher selectivity of the chromatographic method. However, the RadesPhone device and the color analysis methodology with multivariate analysis offer several opportunities for the use of MIP in different systems and analytes, allowing for the development of fast, cost-effective, and easy-to-use methods of analysis. Furthermore, the developed MIP can be prepared to selectively adsorb and improve its sensitivity by obtaining intensely colored compounds. MIP can be supported on various surfaces, such as paper, glass, PET, and others. After immersing the MIP-supported surface in an aqueous solution of the analyte, selective adsorption of a colored compound takes place. Alternatively, the surface can be treated to develop color, followed by direct analysis using the image analysis device. This approach enhances the practicality of the analysis process.

## Figures and Tables

**Table 1:** RGB, HSV, L, and I results obtained for tartrazine standards and soft drink samples.

Sample	R	G	B	I	H	S	V	L
1 ppm	194.5 ± 2.4	174.7 ± 2.1	28.9 ± 23.4	139.1 ± 5.4	52.3 ± 0.6	84.3 ± 12.3	76.0 ± 1.0	43.3 ± 4.0
5 ppm	208.1 ± 1.2	172.5 ± 2.8	3.1 ± 0.1	128.0 ± 1.2	49.3 ± 0.6	98.0 ± 0.0	81.0 ± 0.0	41.0 ± 0.0
10 ppm	206.5 ± 1.3	151.8 ± 0.7	1.9 ± 0.1	120.0 ± 0.1	43.7 ± 0.6	99.0 ± 0.0	80.3 ± 0.6	40.3 ± 0.6
15 ppm	198.9 ± 0.8	140.8 ± 1.3	2.0 ± 0.0	114.0 ± 0.7	42.3 ± 0.6	98.0 ± 0.0	77.3 ± 0.6	39.0 ± 0.0
20 ppm	187.0 ± 1.0	132.7 ± 0.6	0.9 ± 0.1	106.9 ± 0.2	42.0 ± 0.0	99.0 ± 0.0	72.7 ± 0.6	36.3 ± 0.6
25 ppm	186.0 ± 1.0	130.0 ± 1.0	1.3 ± 0.6	105.8 ± 0.2	41.3 ± 0.6	98.7 ± 0.6	72.3 ± 0.6	36.3 ± 0.6
30 ppm	181.0 ± 1.0	125.0 ± 1.0	1.7 ± 0.6	102.6 ± 0.7	41.0 ± 0.0	98.3 ± 0.6	70.3 ± 0.6	36.7 ± 0.6
M1	203.8 ± 1.7	148.0 ± 1.8	1.9 ± 0.1	116.4 ± 1.6	42.5 ± 1.4	97.8 ± 1.6	78.4 ± 2.2	40.5 ± 1.3
M2	192.3 ± 2.0	136.2 ± 1.1	1.5 ± 0.1	111.2 ± 1.6	41.5 ± 1.3	97.3 ± 2.0	74.5 ± 1.3	37.2 ± 1.0
M3	192.4 ± 1.2	137.4 ± 1.5	1.6 ± 0.5	111.1 ± 0.8	40.8 ± 0.8	94.4 ± 1.5	75.4 ± 1.4	37.1 ± 1.0
M4	193.9 ± 1.7	136.9 ± 1.7	1.4 ± 0.3	110.8 ± 0.9	42.9 ± 1.0	97.6 ± 1.4	75.2 ± 1.1	36.3 ± 1.5
M5	205.0 ± 1.1	150.4 ± 1.6	1.6 ± 0.5	117.6 ± 1.4	43.4 ± 0.5	97.3 ± 1.4	80.6 ± 1.3	40.8 ± 1.1

**Table 2:** Summary of R^2^ values according to the studied concentration range.

Range (mg/L)	Number of Components	R^2^ (Calibration)	R^2^ (Validation)
0–30	4	0.980973	0.967121
0–20	5	0.995304	0.967887

**Table 3:** Figures of merit for the PLS model developed for tartrazine by color analysis.

Range (mg/L)	Number of Components	Repeatability (*n* = 10)			Limit of Detection (LOD) (mg/L)
		mg/L	Found (mg/L)	% RSD	
0–20	4	4	4.6	5.3	1.2
		8	7.2	3.3	
		15	14.5	4.6	

**Table 4:** Amount of tartrazine found in soda drinks by the proposed RadesPhone and UHPLC method.

Amount of Tartrazine Found (mg/L, *n* = 3)				
Sample	RadesPhone	UHPLC	Relative Error (%)	RSD (%)
M1	13.6 ± 0.8	12.8 ± 0.2	6.3	5.9
M2	17.4 ± 0.6	19.4 ± 0.3	−10.3	3.5
M3	11.2 ± 0.7	9.6 ± 0.2	16.7	6.3
M4	18.1 ± 0.7	16.8 ± 0.1	7.7	4.6
M5	14.0 ± 0.6	12.6 ± 0.3	15.9	4.3

## Data Availability

Not applicable.
